# Three-dimensional Culturing Method with Decellularized Brain Tissue

**DOI:** 10.14789/ejmj.JMJ25-0009-OT

**Published:** 2025-10-10

**Authors:** RYUSEI ABE

**Affiliations:** 1 Research Institute for Diseases of Old Age, Juntendo University Graduate School of Medicine, Tokyo, Japan; 1 Research Institute for Diseases of Old Age, Juntendo University Graduate School of Medicine, Tokyo, Japan

**Keywords:** chondroitin sulfate, extracellular matrix (ECM), oligodendrocytes, oligodendrocyte precursor cell (OPC), decellularized tissue

## Abstract

We determined the optimal culture conditions for observing the interactions between the extracellular matrix (ECM) structure and oligodendrocyte lineage cells in three-dimensional (3D) decellularized brain tissue.

## Objectives

We determined the suitable culture conditions for observing the interactions between the extracellular matrix (ECM) structure and oligodendrocyte lineage cells in three-dimensional (3D) decellularized brain tissue. We previously developed a 3D culture system based on decellularized brain tissue^1)^ that mimics the complex ECM structure and in vivo conditions.

## Methods

Stem cells were collected from mouse embryos, cultured in NS medium (DMEM, epidermal growth factor (EGF), fibroblast growth factor 2 (FGF2), hepatocyte growth factor (HGF), B27, Penicillin/Streptomycin(Pen/St), and Amphotericin B(AmpB)) for 1 week, and grafted into decellularized tissue to evaluate neural stem cell differentiation in the 3D culture system. Stem cell differentiation into neurons was observed after culturing with differentiation medium (DMEM, Pen/St, AmpB, HGF, and fetal bovine serum (FBS)).

## Results

**Figure 1:**
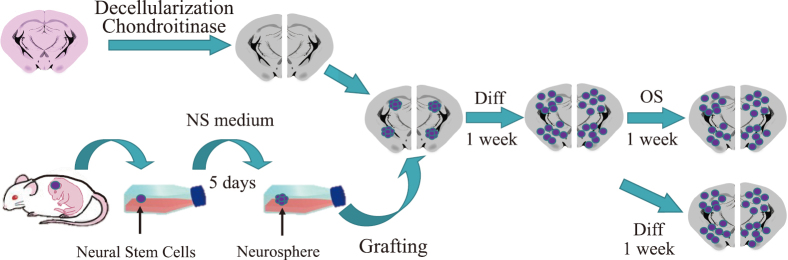
Schematic of the protocol The effect of changing the buffer from differentiation medium (DMEM, Pen/St, AmpB, HGF, and FBS) to OS medium (DMEM, Pen/St, AmpB, B27, HGF, PDGFAA, and FGF2) on stem cell differentiation was investigated. Diff: differentiation medium, OS: OS medium

The neural stem cells predominantly differentiated into astrocytes; no interactions were observed between the ECM and oligodendrocyte lineage cells. The standard neural stem cell culturing protocol was improved to increase oligodendroglia differentiation. The cells were cultured in OS medium (DMEM, Pen/St, AmpB, B27, and FGF2, supplemented with HGF and platelet-derived growth factor AA (PDGFAA)). The differentiation of the stem cells into neurons and oligodendrocytes was observed; however, neural differentiation was limited, and no interaction was observed between the ECM and oligodendrocyte lineage cells. The effect of changing the buffer from differentiation to OS medium on stem cell differentiation was investigated. Figure 1 depicts a schematic of this protocol. Cells were cultured in differentiation medium for one week after grafting, then cultured in OS medium for another week. The results were compared with those of cells cultured in differentiation medium for 2 weeks. Premyelination state was confirmed via immunostaining for differentiation-specific markers. The semiquantitative RT-PCR results demonstrated that switching from differentiation to OS medium 1 week after grafting increased differentiation into neuroblasts, neurons, and oligodendrocytes.

## Conclusions

Switching the buffer from differentiation to OS medium 1 week after grafting provides suitable conditions for investigating the crosstalk between oligodendrocyte differentiation and the structure of the ECM.

## Author contributions

The author read and approved the final manuscript.

## Conflicts of interest statement

The author declares no conflicts of interest associated with this manuscript.
